# Mutagenicity and Genotoxicity Assessment of *Leuconostoc lactis* DMLL10 Isolated from Kimchi

**DOI:** 10.4014/jmb.2405.05025

**Published:** 2024-08-05

**Authors:** Heejung Park, Seoyeon Lee, Sojeong Heo, Do-Won Jeong

**Affiliations:** 1 Department of Foodservice Management and Nutrition, Sangmyung University, Seoul 03016, Republic of Korea; 2 Department of Food and Nutrition, Dongduk Women’s University, Seoul 02748, Republic of Korea

**Keywords:** *Leuconostoc lactis* DMLL10, genotoxicity, bacterial reverse mutation assay, chromosomal aberration assay, mammalian micronucleus test

## Abstract

*Leuconostoc lactis* DMLL10 is a microorganism specific to kimchi fermentation. In this study, we sought to evaluate the toxicity of this strain, which was newly isolated from kimchi, to determine its safety as a food ingredient. Bacterial reverse mutation assay, chromosomal aberration assay, and mammalian cell *in vitro* micronucleus assay were performed to assess the genetic toxicity of *Leu. lactis* DMLL10. The strain did not induce mutagenicity in *Salmonella typhimurium* TA98, TA100, TA1535, TA1537, or *Escherichia coli* WP2uvrA, with or without metabolic activation of S9 mixture. The oral administration of *Leu. lactis* DMLL10 also did not significantly increase the number of micronucleated polychromatic erythrocytes, or the mean ratio of polychromatic to total erythrocytes. Additionally, *Leu. lactis* DMLL10 did not cause a significant chromosomal aberration in CHU/IL cells in the presence or absence of S9 activation. Therefore, *Leu. lactis* DMLL10 can be suggested as a functional food ingredient with reliability and safety.

## Introduction

Kimchi is a traditional Korean dish made by fermenting vegetables, primarily kimchi cabbage, with various seasonings. Kimchi includes various nutrients, such as vitamin K, vitamin C, folate, and potassium, which are essential for overall health [1]. It holds a significant place in Korean cuisine, providing essential nutrients, as well as numerous health advantages. In recent years, kimchi has gained popularity beyond Korea and is now enjoyed worldwide. Its unique flavor profile, health benefits, and versatility have contributed to its global appeal. As a fermented food, kimchi undergoes a process whereby beneficial bacteria, known as probiotics, break down the sugars in the vegetables. These probiotics can have positive effects on gut health by promoting the growth of good bacteria in the digestive system [2-4]. The probiotics present in kimchi can support a balanced gut microbiome, enhancing digestion and nutrient uptake. Alongside the essential nutrients in kimchi, these probiotics bolster the immune system by fostering a healthy balance of the gut bacteria [5-7]. Meanwhile, a fortified immune system plays a crucial role in preventing infection and illness.

The primary microorganisms involved in kimchi fermentation are bacteria, the most notable of which are lactic acid bacteria (LAB) [8]. The specific strains of LAB can vary, but in general, they belong to the genera *Lactobacillus*, *Leuconostoc*, or *Weissella*. *Leuconostoc lactis* is a kimchi-oriented microorganism that is often present in the early stages of fermentation, and contributes to flavor and texture development [9]. It produces various compounds, including carbon dioxide, which creates the characteristic bubbles in well-fermented kimchi. Leuconostoc (Leu.). lactis is already listed in the Qualified Presumption of Safety (QPS) and International Dairy Federation (IDF), and is recognized as a non-pathogenic microorganism [10-14].

The probiotic strains derived from kimchi exert numerous physiological activities, with various strains reported, including *Lactiplantibacillus (Lab.) plantarum* KU15120 [15], *Limosilactobacillus (Lib.) fermentum* JNU532 [16], *Enterococcus faecium* FC-K [17], *Pediococcus acidilactici* PMC48 [18], and *Lentilactobacillus (Leb.) buchneri* KU200793 [19]. Most kimchi-derived probiotics exhibit antioxidant properties [15, 16, 19], with *Lab. plantarum* KU15120 showing anti-obesity effects, while *Lib. fermentum* JNU532 demonstrates whitening effects. *Enterococcus faecium* FC−K is associated with anti-allergic properties, while *Leb. buchneri* KU200793 shows observed neuroprotective effects. The diverse physiological effects of these kimchi-derived probiotics point to their own considerable potential utility as functional ingredients themselves.

In a previous study, we isolated a novel *Leu. lactis* strain DMLL10 from kimchi, and reported its functionality [20]. In addition, *Leu. lactis* DMLL10 as a starter candidate showed probiotic attributes in kimchi. These include resistance to acid and bile salts, absence of genes transferring antibiotic resistance, and absence of hemolytic activity. However, research on its toxicity remains insufficient. In this study, we performed bacterial reverse mutation assay, chromosomal aberration assay, and mammalian micronucleus test at concentrations of up to maximum, to determine the genetic toxicity of *Leu. lactis* DMLL10.

## Materials and Methods

### Sample Preparation

*Leu. lactis* DMLL10 from kimchi was selected as a novel starter candidate and subjected to *in vitro* experiments and genomic analysis [20]. The strain was used as a lyophilized powder with a concentration of 6.8 × 10^10^ CFU/g. It was grown in 500 L of food-grade media (2% glucose, 1% yeast extract, 1% soy peptone, 0.5% CH_3_COONa, 0.2%K_2_HPO_4_, 0.01% MgSO_4_, 0.005% MnSO_4_) at 30°C for 18 h. After incubation, the broth was centrifuged at 7,500 ×*g* for 10 min, the supernatants were discarded, and the cell pellets were freeze-dried and stored at −20°C until use.

### Experiments

This study was conducted in compliance with the Good Laboratory Practice regulations (Biotoxtech, Republic of Korea) to investigate genotoxicity of strain *Leu. lactis* DMLL10, and determine whether it directly damages DNA or chromosomes, leading to morphological changes or functional abnormalities. The genotoxicity testing consisted of microbial reverse mutation assay, in vivo micronucleus assay using bone marrow cells, and chromosomal aberration assay. All experiments were conducted with approval from the Biotoxtech Animal Ethics Committee (Approval no. 220685), under the Animal Protection Act (Law no. 4379).

### Bacterial Reverse Mutation Assay

For microbial reverse mutations, experiments were conducted in accordance with the Organization for Economic Cooperation and Development (OECD) guidelines for the testing of chemicals [21]. The mutagenic properties were determined using four histidine-requiring strains of *Salmonella typhimurium* (TA98, TA100, TA1535, and TA1537), and one tryptophan-requiring strain of *Escherichia coli* (WP2 uvrA). The strains used in this study were subjected to confirmation tests, such as amino acid requirements, UV sensitivity, rfa mutation, and R–factor maintenance, in accordance with Maron and Ames’ methods [22], to ensure that the genetic characteristics of the test strains were well preserved, before proceeding with the experimentation. The solvent capable of dissolving the test substance, Dulbecco’s phosphate-buffered saline (DPBS, Lonza Walkersville Inc., USA), was used as the positive control. The mutagenic positive controls used for inducing microbial reverse mutations in each strain included 2–nitrofluorene (2−NF, Sigma–Aldrich, USA), sodium azide (NaN_3_, Sigma–Aldrich), 9–aminoacridine (9−AA, Sigma–Aldrich), 4–nitroquinoline 1–oxide (4NQO, Sigma–Aldrich), and 2–aminoanthracene (2−AA, Sigma–Aldrich). Testing was conducted through preincubation, with and without metabolic activation (+S9 mix and −S9 mix), utilizing the metabolic activation system (S9 mix) purchased from MOLTOX™ (Molecular Toxicology Inc., USA). Based on the results of the concentration determination test, the main test was conducted at five concentration levels (313, 625, 1,250, 2,500, and 5,000 μg/plate) under metabolic activation and nonactivation conditions. Under non-metabolic activation conditions, 100 μl of each dose of the test substance, and negative and positive control substances, were placed into separate tubes, followed by the addition of 500 μl of 0.1 mol/l phosphate buffer (pH 7.4) and 100 μl of each bacterial suspension. The tubes were then incubated with shaking for 20 min at 37°C and 90 rpm. After incubation, 2 ml of *Salmonella* top agar was added to strains TA98, TA100, TA1535, and TA1537, and 2 ml of *E. coli* top agar was added to strain WP2uvrA. The mixtures were vortexed, poured onto minimal glucose agar plates, and allowed to solidify at room temperature (RT). Under metabolic activation conditions, 500 μl of S9 mix was added, instead of 500 μl of 0.1 mol/l phosphate buffer (pH 7.4). All other procedures remained the same. After solidification of the top agar, the plates were inverted, and the incubated at 37°C in a bacteriological incubator (DK−LI020−P, Daiki Scientific Co., Ltd., Republic of Korea) for 48 h. Any precipitation of the test substance during treatment and colony count measurement was observed visually and recorded. After incubation, the revertant colonies were counted by automated colony counter (ProtoCOL3, SYNBIOSIS, UK), or manual counting. Manual counting was performed if the automated counting was deemed inaccurate.

### Chromosomal Aberration Assay

The experiments to assess the induction of chromosomal aberrations were conducted according to the OECD guidelines [23]. The Chinese hamster lung cell line CHL/IU was obtained from the American Type Culture Collection (USA), and utilized to evaluate chromosomal aberration induction. In the presence of metabolic activation (+S9 mix), Benzo[a]pyrene (B[a]P, Sigma–Aldrich) was used as positive control, while in the absence of metabolic activation (−S9 mix), mitomycin C (MMC, Sigma–Aldrich) was used as positive control. DPBS was used as the negative control. CHL/IU cells were cultured using Eagle’s minimum essential medium (Lonza Walkersville Inc., USA) with 10% fetal bovine serum (FBS, Gibco, USA). In a 25-T culture flask, 5 × 10^4^ cells/ml was added to the test substance. Approximately 22 h after the initiation of test substance application for all flasks, 100 μl of colcemid (0.2 μg/ml) was added to each flask, and cells were collected after 2 h incubation. After centrifugation and removal of the supernatant, the cells were treated with a hypotonic solution of 75 mM KCl at 37°C for 20 min. After adding 1 ml of cold fixative (methanol:acetic acid = 3:1), the sample was centrifuged at 1,000 ×*g* for 5 min, and the supernatant was removed. Then, 5 ml of cold fixative was added, and the sample was centrifuged at 2,000 ×*g* for 5 min to fix the cells. This fixation process was repeated once. After floating the cells in a small amount of cold fixative, one drop was placed on each of two microscope slides to create one sample slide. The slides were air-dried, and a code number was assigned to each slide. They were then stained with 3% Giemsa stain for 20 min, washed with distilled water, dried, and mounted with a mounting medium (Entellan New, Germany). Sample slides were observed following the sequence of continuous processing after short-term treatment. For chromosomal observations, three doses were set for each processing method, with each dose allowing for the observation of at least 300 mid-metaphase cells. These cells were observed under a microscope (600× magnification, BX51, Olympus, Japan). Chromosomal abnormalities were classified into structural, numerical, and other abnormalities. Structural abnormalities included chromatid breaks (ctb), chromatid exchanges (cte), chromosome breaks (csb), chromosome exchanges (cse), chromatid gaps (ctg), chromosome gaps (csg), and fragmentations (frg). If multiple gaps and breaks were observed in one mid-metaphase cell, it was recorded as fragmentation. Gaps were defined as non-staining regions narrower than the width of a chromatid. Additionally, numerical abnormalities, such as polyploidy (pol) and endoreduplication (end), were observed. Cells with one or more of these abnormalities were counted as one abnormal cell, and the types were recorded individually. For gaps, abnormal cells were recorded separately, depending on whether a gap was observed. “Other” included abnormalities not classified as structural or numerical, and the type and number of these abnormalities were recorded. For the frequency of cells with chromosomal abnormalities (excluding gaps), the following conditions were considered positive if all were met: the frequency of cells with chromosomal abnormalities would significantly increase compared to the negative control group in one or more doses; there would be a dose-dependent relationship with the increase; and the frequency of cells with chromosomal abnormalities would exceed the control limit of historical control data for the negative control group. Otherwise, they were deemed negative.

### Mammalian Micronucleus Test

A total of twenty-four Sprague-Dawley male rats (7 weeks old, 199.5 − 220.3 g) were used in this study. The animals were purchased from Samtako Bio Korea Co., Ltd. (Republic of Korea), and acclimated to the animal housing facility for 6 days, before being utilized in the experiment. The animal facility maintained a temperature of (19.0 − 25.0°C), relative humidity of (40.0 – 70%), a 12 h light–dark cycle (lights on at 07:00, and off at 19:00), lighting intensity ranging from 150 to 300 Lux. The experimental animals were housed in stainless cages (260 mm W × 350 mm D × 210 mm H), with no more than 3 mice per cage. A certified irradiated solid diet for experimental animals (Teklad-certified irradiated global 18% protein rodent diet 2918C, Envigo RMS, Inc., USA) and water was provided for ad libitum consumption. *Leu. lactis* DMLL10 was administered orally once a day, with a total of 2 administrations at 24 h interval. The dose volume was 10 ml/kg, based on the body weight measured on the day of administration. The study consisted of a solvent control group (DPBS), 3 treatment groups of (1,563, 3,125 and 6,250 mg/kg body weight/day), and a positive control group (Cyclophosphamide 20 mg/kg bw/day), forming a total of 5 groups (Table 1). The animals were euthanized by CO_2_ gas anesthesia 24 h after the second dose of the test substance. The body weight was measured on the day of dosing initiation and bone marrow collection. The femur was excised, and the muscular tissue was thoroughly removed. Both ends were cut with scissors, and 0.5 ml of phosphate-buffered saline (PBS, Lonza Walkersville Inc.) was perfused to collect bone marrow cells. To the bone marrow cell suspension, 0.5 ml of 10% neutral-buffered formalin was added for fixation for 5 min. After fixation, centrifugation was performed for 5 min at 4°C and 1,000 ×*g* (Micro17TR, Hanil Science Industrial, Republic of Korea) to remove the supernatant. Subsequently, 0.3 ml of 10% neutral-buffered formalin was added to the precipitated bone marrow cells for floating, followed by filtration through a cell strainer into a storage tube. The storage tube was labeled with a code number and kept at RT until observation. The floated bone marrow cell suspension was dropped onto a coverslip, and a slide glass coated with 20 μl of 0.05% acridine orange was placed on top to prepare observation slides. The prepared slides were observed under a microscope. The occurrence rate of micronucleated polychromatic erythrocytes (MNPCE) per 4,000 polychromatic erythrocytes (PCE) for each was determined. From the observation results, the ratio of MNPCE to total erythrocytes in the test substance group was examined to determine if it exceeded 20% of the negative control group. As an indicator of bone marrow cell proliferation inhibition, the ratio of MNPCE to total erythrocytes was calculated by observing 500 total erythrocytes per individual.

The criteria for a positive result included a statistically significant increase in the frequency of MNPCE compared to the negative control group at one or more doses, along with a dose-dependent increase and a frequency of MNPCE surpassing the control limit, based on historical control data from the negative control group. Otherwise, the result was considered negative.

### Statistical Analysis

Regarding the measurement of revertant colony counts in the bacterial reverse mutation, the actual values were recorded. Statistical analyses were conducted using SAS version 9.4 (SAS Institute Inc., USA). For the frequency of cells with chromosomal abnormalities (excluding gaps), Fisher’s exact test was conducted to assess the significance between the negative control group and the test substance group, as well as between the negative control group and the positive control group (*p* < 0.05). In the micronucleus test, the frequency of MNPCE was assessed using the Mann–Whitney U test to determine significance at *p* < 0.05. Cochran–Armitage trend test was employed to assess dose-dependency among the test substance groups (*p* < 0.05). For comparisons between the negative control group and positive control group, Student’s *t*-test was performed to determine the significance (*p*<0.05).

## Results

### Bacterial Reverse Mutation Assay

In the test substance groups, regardless of metabolic activation, the revertant colony counts for each strain at all doses did not exceed twice those of the negative control group. The revertant colony counts in the positive control groups for each strain were significantly increased by more than twice, compared to the negative control group. No growth inhibition or precipitation caused by the test substance was observed at any dose for both, with and without metabolic activation across all strains. The test demonstrated reproducibility in the results of genetic mutagenicity, and identified doses where no growth inhibition was observed, ensuring at least a fourfold margin. The average revertant colony counts of both negative and positive control groups fell within the range of historical control data (Table 2), and the revertant colony counts of positive control groups for each strain were significantly increased by more than twice, compared to the negative control group. Furthermore, no contamination by stray bacteria was detected, indicating that the test was conducted appropriately. Based on the above results, we concluded that under the conditions of this test, the test substance, kimchi-derived probiotic strain *Leu. lactis* DMLL10, can be considered non-mutagenic.

### Chromosomal Aberration Assay

Table 3 presents the results of the chromosomal aberration assay. In the short-term treatment method, at concentrations where metabolic activation was absent, 87.4% or more of cells were observed with concentrations of 0, 78, 156, 313, and 625 μg/ml, while at concentrations where metabolic activation was present, 87.7 % or more of cells were observed with concentrations of 0, 78, 156, 313, and 625 μg/ml. In the continuous treatment method, at concentrations where metabolic activation was absent, 82.6% or more of cells were observed with concentrations of 0, 78, 156, 313, and 625 μg/ml. There was no statistically significant difference in the frequency of cells with chromosomal abnormalities compared to the negative control group in both the absence and presence of metabolic activation in the short-term treatment method, as well as in the absence of metabolic activation in the continuous treatment method. However, in each treatment group, there was a statistically significant increase in the frequency of cells with structural abnormalities, compared to the negative control group (*p* < 0.01).

### Mammalian Micronucleus Test

**Fig. 1:**
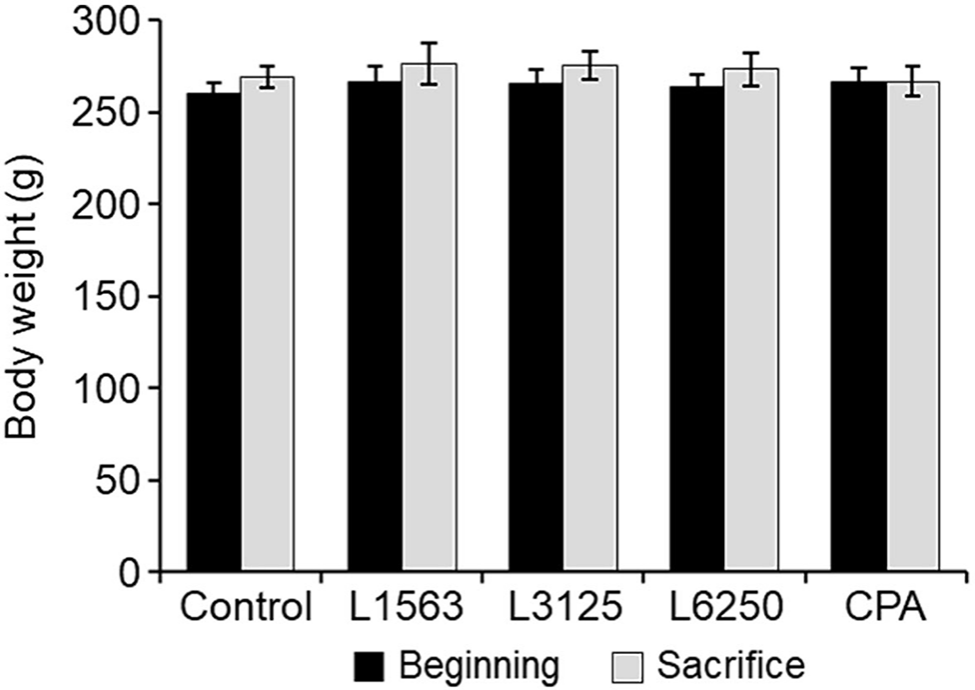
Body weight of experimental animals by group. L1563, 1,563 mg/kg body-weight (bw)/day *Leuconostoc lactis* DMLL10-administered group; L3125, 3,125 mg/kg bw/day *Leu. lactis* DMLL10-administered group: L6250, 6,250 mg/kg bw/ day *Leu. lactis* DMLL10-administered group; CYP: cyclophosphamide (positive control).

During the observation period, no general symptoms attributable to the test substance were observed in any dose group. Additionally, statistically significant weight changes compared to the negative control group were not observed in any dose group (Fig. 1). The frequency of MNPCE, as well as the ratio of PCE to total erythrocytes, did not show statistically significant differences, compared to the negative control group, in any dose group.

However, in the positive control group (CPA), a statistically significant increase in the frequency of MNPCE, compared to the control group, was observed (*p* < 0.01). The ratio of PCE to total erythrocytes did not show statistically significant differences, compared to the negative control group (Table 4).

In the negative control group, the frequency of MNPCE fell within the control range of the historical control data, and within the 95% range of the historical control data. Additionally, in the positive control group, the frequency of MNPCE fell within the control range of the historical control data, and showed a statistically significant increase, compared to the negative control group. The number of administered and observed cells was adequate, and the highest dose was determined according to the dose-setting criteria, thus the test was considered to be conducted under appropriate test conditions. Based on the above results, kimchi probiotic *Leu. lactis* DMLL10 was determined to be negative for micronucleus induction in Sprague–Dawley rat bone marrow cells under the conditions of this study.

## Discussion

Probiotics have garnered significant attention for their role in modulating the intestinal microbiota and ability to restore the abundance of specific gut microorganisms crucial for human health [24]. This has led to the emergence of the concept of microbial medicine, particularly in addressing conditions like obesity and metabolic syndrome [25], thereby broadening the scope of probiotics in preventive medicine and the realm of functional foods. Functional foods have become a focal point in both social discourse and scientific inquiry. These are foods fortified with additional health-promoting ingredients or naturally rich in compounds that offer specific health benefits beyond basic nutrition. However, the development of functional food products necessitates a multi-faceted approach; it is essential to adequately incorporate consumer preferences, but more importantly, scientific efficacy validation, and above all, prioritized safety assessments must be conducted. Therefore, before marketing or consumption, the safety and efficacy of functional foods must be ensured.

*Leu. lactis* DMLL10 was first isolated by the researchers, and is intended to be commercialized as a starter or functional food ingredient in processed foods. Therefore, its safety as a food ingredient must be tested according to OECD standard procedures. In this research, the bacterial reverse mutation assay, chromosomal aberration assay, and mammalian micronucleus test were conducted as part of the genotoxicity testing.

Genotoxicity refers to the properties of chemical agents or physical agents that damage the genetic information within a cell, leading to mutations or other genetic alterations. Genotoxic substances can cause DNA damage directly or indirectly, leading to cancer [26]. Therefore, genotoxicity tests are often conducted as part of regulatory requirements to evaluate the safety of chemicals and products. The results demonstrated that *Leu. lactis* DMLL10 did not elicit any indications of reverse mutation, chromosomal abnormality, or micronuclei formation across all test systems. Specifically, no mutagenic potential was observed in *Salmonella typhimurium* TA98, TA100, TA1535, TA1537, or *Escherichia coli* WP2*uvrA*, with or without metabolic activation by the S9 mixture. Additionally, no significant induction of chromosomal aberrations was noted in CHU/IL cells under both activated and non-activated metabolic conditions. Furthermore, oral administration of *Leu. lactis* DMLL10 did not result in a significant increase in micronucleated polychromatic erythrocytes, or the mean ratio of polychromatic to total erythrocytes. Our findings collectively affirm the non-genotoxic nature of *Leu. lactis* DMLL10 across various test conditions. The results also provide valuable insight into the safe and efficacious utilization of *Leu. lactis* DMLL10 as a natural product for future development and application.

In conclusion, for the bacterial reverse mutation assay, *Leu. lactis* DMLL10 did not induce growth inhibition or precipitation at any dose, regardless of metabolic activation. No abnormalities were observed in the chromosomal aberration assay. In the micronucleus test, there were no significant differences in the frequency of PCE or the total ratio of erythrocytes with micronuclei, compared to the negative control group, across all doses. Considering these results collectively, under the conditions of this study, *Leu. lactis* DMLL10 can be regarded as a safe and reliable functional food ingredient.

## Figures and Tables

**Table 1:** Group assignment and treatment conditions in the mammalian micronucleus test.

Group	Sex	N	Volume (ml/kg)	Dosage (mg/kg body-weight/day)
Control	Male	5	10	0
L1563^1)^	Male	5	10	1,563
L3125^2)^	Male	5	10	3,125
L6250^3)^	Male	5	10	6,250
CYP^4)^	Male	5	10	20

^1)^L1563, 1,563 mg/kg body-weight (bw)/day *Leuconostoc lactis* DMLL10-administered group; ^2)^L3125, 3,125 mg/kg bw/day *Leu. lactis* DMLL10-administered group; ^3)^L6250, 6,250 mg/kg bw/day *Leu. lactis* DMLL10-administered group; ^4)^CYP, cyclophosphamide (positive control).

**Table 2:** Mutagenicity of *Leuconostoc lactis* DMLL10 without and with metabolic activation in the bacterial reverse mutation assay.

Mutation	Tester strain	Samples	Dose (μg/plate)	Colonies/plate	
				without S9 mix	with S9 mix
Substitutio n mutation	*Salmonella typhimurium* TA100	DMLL10	0	122.5 ± 2.00	134.0 ± 1.00
			313	123.0 ± 1.50	136.0 ± 1.50
			625	127.5 ± 2.00	135.0 ± 3.00
			1,250	133.5 ± 2.00	137.5 ± 2.00
			2,500	145.0 ± 4.00	145.5 ± 2.00
			5,000	154.5 ± 2.50	149.5 ± 2.00
		Sodium azide	1.5	648.0 ± 14.00	
		2–Aminoanthracene	2		882.5 ± 28.00
	*Salmonella typhimurium* TA1535	DMLL10	0	11.5 ± 1.00	11.0 ± 1.50
			313	11.5 ± 1.00	10.0 ± 1.00
			625	10.0 ± 1.00	10.0 ± 1.00
			1,250	12.5 ± 1.00	11.5 ± 1.00
			2,500	11.0 ± 1.00	13.0 ± 1.00
			5,000	12.0 ± 1.50	14.5 ± 1.00
		Sodium azide	1.5	573.0 ± 16.00	
		2–Aminoanthracene	3		185.0 ± 9.50
	*Escherichia coli* WP2 urvA	DMLL10	0	28.0 ± 1.00	31.5 ± 2.00
			313	28.5 ± 1.00	32.0 ± 2.00
			625	29.0 ± 1.00	32.5 ± 1.00
			1,250	30.0 ± 1.00	34.0 ± 1.50
			2,500	31.0 ± 1.50	33.5 ± 1.50
			5,000	32.5 ± 1.50	36.0 ± 2.00
		4–Nitroquinoline 1-oxide	0.3	340.7 ± 20.00	
		2–Aminoanthracene	10		555.0 ± 21.00
Frameshift mutation	*Salmonella typhimurium* TA98	DMLL10	0	18.0 ± 1.50	31.5 ± 1.50
			313	18.5 ± 1.50	31.5 ± 1.50
			625	19.0 ± 1.00	29.0 ± 1.00
			1,250	19.5 ± 1.50	32.0 ± 1.00
			2,500	20.5 ± 2.00	31.5 ± 1.50
			5,000	22.0 ± 1.50	33.0 ± 1.00
		2–Nitrofluorene	5	592.0 ± 16.00	
		2–Aminoanthracene	1		369.0 ± 18.50
	*Salmonella typhimurium* TA1537	DMLL10	0	10.0 ± 1.50	12.5 ± 1.00
			313	9.5 ± 1.00	12.5 ± 1.50
			625	9.0 ± 1.50	14.0 ± 1.00
			1,250	10.5 ± 1.50	12.5 ± 1.50
			2,500	11.5 ± 1.00	14.0 ± 1.00
			5,000	11.5 ± 1.00	15.5 ± 1.00
		9–Aminoanthracene	80	607.0 ± 21.00	
		2–Aminoanthracene	3		180.5 ± 8.00

**Table 3:** Chromosomal aberration incidence of *Leuconostoc lactis* DMLL10.

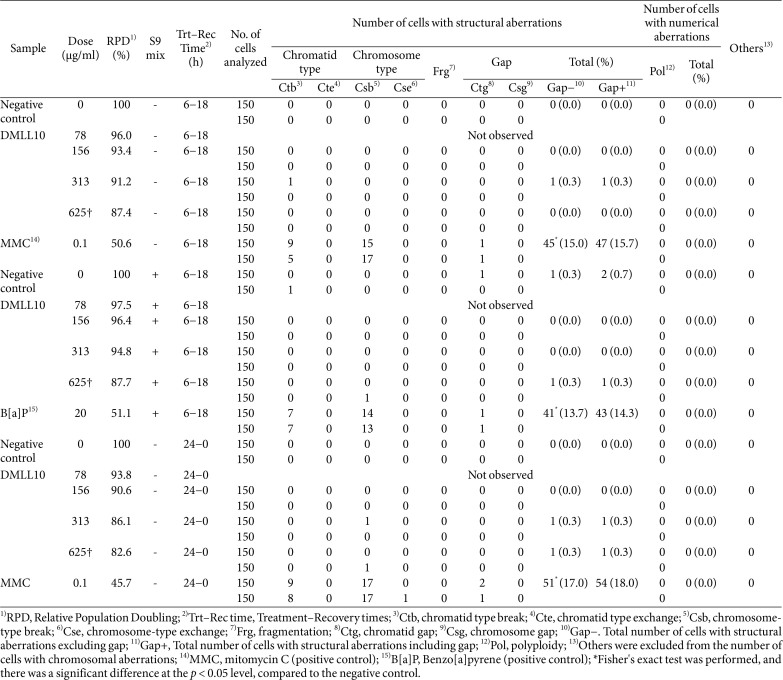

**Table 4:** Effect of *Leuconostoc lactis* DMLL10 on the formation of micronucleus in rat.

Samples	Dose mg/kg bw/day	Hours after dosing	PCE^1)^/500 RBC (%)	MNPCE^2)^/4000 PCE (%)
Control	0	24	32.4 ± 0.85	0.030 ± 0.011
DMLL10	1,563	24	32.1 ± 0.18	0.025 ± 0.018
	3,125	24	32.2 ± 0.64	0.030 ± 0.011
	6,250	24	32.2 ± 0.79	0.035 ± 0.014
Cyclophosphamide (Positive control)	20	24	32.4 ± 0.73	4.44 ± 0.113*

^1)^PCE, polychromatic erythrocyte; ^2)^MNPCE, micronucleated polychromatic erythrocyte; *Mann–Whitney U test was performed, and there was a significant difference at the *p* < 0.01 level, compared to the negative control.
